# Traumatic appendicitis is probably not real: an illustrative analysis of coincidental occurrences in nature

**DOI:** 10.1136/tsaco-2023-001093

**Published:** 2023-03-15

**Authors:** David N Naumann, Tom Barker

**Affiliations:** 1 Department of Emergency General Surgery and Trauma, University Hospitals Birmingham NHS Foundation Trust, Birmingham, UK; 2 Institute of Inflammation and Ageing, University of Birmingham, Birmingham, UK; 3 Academic Department of Military Surgery and Trauma, Royal Centre for Defence Medicine, Birmingham, UK; 4 Department of Vascular Surgery, University Hospitals Birmingham NHS Foundation Trust, Birmingham, UK

**Keywords:** Appendicitis, Models, Statistical, Multiple Trauma, abdominal injuries

## Abstract

There have been sporadic case reports describing ‘traumatic appendicitis’ (acute appendicitis occurring following injury) for almost a hundred years. Although this might seem to be an interesting and rare diagnosis for the journal reader, both appendicitis and trauma are very common, and their occurrence together may only give the illusion of causality. Indeed, such a diagnosis may not even exist. We provide an illustration of the statistical phenomenon of coincidental occurrences in nature using a computer simulation of traumatic appendicitis in the UK population. In our simulation, there are enough cases of traumatic appendicitis every 2 years to 3 years to account for the entire global literature on the topic. We suggest that unless there is a credible pathological process reported with demonstrable causality, further case reports of traumatic appendicitis need to have robust justification.

According to popular folklore, the famous magician Harry Houdini died following a perforated appendicitis—supposedly from a punch to the abdomen. Traumatic appendicitis is a recurring topic in medical literature, described in the literature as far ago as 1927.^1^ More recently, case reports every few years describe traumatic appendicitis following a diverse range of accidents and misadventures from cliff dives to camel kicks, as illustrated in table 1. Although ‘traumatic appendicitis’ might be an appealing diagnosis to beguile the surgical journal reader, both appendicitis and trauma are very common and will therefore occur in a population enough to give the illusion of causality. This logical fallacy of event A occurring before event B and therefore being the cause of event B is referred as post hoc ergo propter hoc (literally ‘after this, therefore because of this’).

**Table 1:** Selection of English language case reports in the 20th century that describe ‘traumatic appendicitis’

Year	Study	Age	Sex	Type of trauma	Time interval (trauma to diagnosis)
2002	Ramesh *et al*^6^	11	M	Bicycle handlebar	2 days
2002	Hagger *et al*^7^	60	M	Fall from ladder	3 days
2009	Amir *et al*^8^	10	M	Blunt perineal trauma	2 hours
2009	Derr and Goldner^9^	41	M	Cliff dive	2 days
2010	Attala *et al*^10^	53	M	Struck by car door	7 hours
2010	Toumi *et al*^11^	11	M	Trampoline accident	3 days
2013	Goldman *et al*^12^	11	M	Wrestling	<24 hours
2013	Bouassria *et al*^13^	24	M	Stabbing	24 hours
2012	Paschos *et al*^14^	17	F	Bicycle handlebar	24 hours
2014	Ahmed *et al*^15^	12	M	Corner of a table	2 days
2017	Cobb^16^	17	M	RTC	24 hours
2017	Khilji *et al*^17^	43	M	RTC	<24 hours
2020	Zvizdic *et al*^18^	7	M	Horse hoof	17 hours
2021	Toffaha *et al*^19^	35	M	Camel kick	2 days
2021	Chalh *et al*^20^	8	M	Fall playing soccer	24 hours
2022	Salinas-Castro *et al*^21^	14	F	Soccer ball	6 hours

F, female; M, male; RTC, road traffic collision.

Here we provide an illustration of the statistical phenomenon of coincidental occurrences in nature using traumatic appendicitis in the UK population. A computer model programmed using Perl V. 5.32 (https://www.perl.org) was used to simulate the number of appendicitis and traumatic events and their temporal relationship in the UK population in a year. The model assumes a UK population of 67 100 000 people^2^ and a rate of appendicitis of 151 per 100 000.^3^ The incidence of trauma, especially of a trivial and reported nature, is unknown, so we ran the model for an incidence of trauma of 1:1 (ie, 1 trauma in 1 year), and subsequently in ratios of 1:10, 1:100, 1:1000, and 1:10 000.

## Model methodology

For each individual in the model, a random number between 1 and 100 000 was generated, and if that number was ≤151 then that individual had appendicitis in that year. Similarly, to test for trauma, a random number was generated between one and the trauma probability (1:1, 1:10, 1:100, 1:1000, and 1:10 000). If the number was one then that individual had a traumatic event that year. For individuals with both appendicitis and trauma events in the same year, two random numbers between 1 and 365 were generated (simulating the day of the year the appendicitis and the trauma occurred). If the day of the trauma fell within 3 days before the appendicitis in that year, that case was counted as a case of traumatic appendicitis. For individuals with appendicitis in the first 3 days of the year but no trauma before that, a further test was performed for trauma in that individual on the previous year using the methodology described earlier. A case of traumatic appendicitis was counted if the simulation produced a trauma at the end of the previous year with in 3 days of the appendicitis in the subsequent year. This model was then run 1000 times to produce summary statistics. The code used for the model is shown in online supplemental file 1.

10.1136/tsaco-2023-001093.supp1 Supplementary data

## Model interpretation

Summary statistics for the data produced by the model are shown in table 2. At a trauma frequency of 1:10 000, there were 10.8% of runs that produced cases of traumatic appendicitis, and for a trauma frequency of 1:1000, it was 67.5% of runs. The incidence of major trauma and blunt abdominal trauma at the major trauma centres in one region of the UK has been reported as between 5401/6 000 000 (approximately 1 in 1,000) and 2793/6 000 000 (approximately 1 in 2000),^4^ which is within the range of the modelled statistics. However, given the trivial nature of some of the injuries reported in some cases reports, the relevant trauma frequency can be reasonably assumed to be much higher. At a trauma frequency of 1:100, the UK population alone would randomly generate enough cases of traumatic appendicitis every 2 years to 3 years to account for the entire global literature on this topic.

**Table 2:** Predictive model of the number of expected diagnoses of ‘traumatic appendicitis’ according to the incidence of trauma per year in the UK and 1000 runs of the computer model.

Trauma incidence	Traumas (n)*	With appendicitis (n)*	Traumatic appendicitis*	% runs of model with traumatic appendicitis cases
1 in 1	67 100 000 (67 100 000–67 100 000)	101 304 (100 236–102 633)	1109 (983–1211)	100
1 in 10	6 709 977 (6 703 141–6 717 497)	101 296 (100 311–102 223)	111 (80–156)	100
1 in 100	670 989 (667 466–673 742)	101 327 (100 450–102 404)	11 (1–23)	100
1 in 1000	67 111 (66 313–67 972)	101 325 (100 273–102 265)	1 (0–7)	67.5
1 in 10 000	6709 (6488–6978)	101 313 (100 263–102 140)	0 (0–3)	10.8

*Summarised as median with ranges in parentheses.

Our model also demonstrates another common cognitive bias: the clustering illusion.^5^ Looking at the data for the 1:10 000 trauma frequency, results demonstrated that 89.2% of runs generate no cases of traumatic appendicitis, but in 10.8% of runs, there are clusters of up to three cases. Clustering is a normal phenomenon in small samples of random and pseudorandom data but is frequently interpreted to have significance due to a tendency for us to see patterns in random events. The prevalence, as well as a lack of awareness of these fallacies, is more relevant than ever in the data era and the age of the internet, and has been brought into sharp focus with COVID-19 and the relationship between vaccinations and potential adverse reactions.

While our model cannot not disprove the existence of traumatic appendicitis, it provides a plausible and demonstrable explanation for the phenomenon and an illustrative example of the post hoc fallacy. Unless a robust argument can be made for a legitimate pathological process, with demonstration of causality, justification is required for any further case reports about traumatic appendicitis. Those interested in scientific enquiry may wish to avoid perpetuation of logical fallacies in a world that needs more clarity, not less.

## References

[1] Behan RJ. Traumatic appendicitis. Ann Surg 1927;85:263–8. 10.1097/00000658-192702000-00011 17865621 PMC1399279

[2] Office for National Statistics. Population estimates for the UK, england and wales scotland and northern ireland. Available: www.ons.gov.uk/peoplepopulationandcommunity/populationandmigration/populationestimates/bulletins/annualmidyearpopulationestimates/mid2020

[3] Ferris M, Quan S, Kaplan BS, Molodecky N, Ball CG, Chernoff GW, Bhala N, Ghosh S, Dixon E, Ng S, et al. The global incidence of appendicitis: a systematic review of population-based studies. Ann Surg 2017;266:237–41. 10.1097/SLA.0000000000002188 28288060

[4] Pande R, Saratzis A, Winter Beatty J, Doran C, Kirby R, Harmston C. Contemporary characteristics of blunt abdominal trauma in a regional series from the UK. Ann R Coll Surg Engl 2017;99:82–7. 10.1308/rcsann.2016.0223 27490986 PMC5392782

[5] Kahneman D, Tversky A. Subjective probability: a judgment of representativeness. Cognitive Psychology 1972;3:430–54. 10.1016/0010-0285(72)90016-3

[6] Ramesh G, Ho PW, Ng KL, Jegan T. Appendicitis following blunt abdominal trauma. Med J Malaysia 2002;57:123–4. 14569731

[7] Hagger R, Constantinou J, Shrotria S. Acute appendicitis after a fall from a ladder: a traumatic aetiology? Emerg Med J 2002;19:366–7. 10.1136/emj.19.4.366 12101166 PMC1725897

[8] Amir A, Amir L, Waisman Y. Acute appendicitis after a blunt perineal trauma: an illustrative case. Pediatr Emerg Care 2009;25:184–5. 10.1097/PEC.0b013e31819a8a66 19287277

[9] Derr C, Goldner DE. Posttraumatic appendicitis: further extending the extended focused assessment with sonography in trauma examination. Am J Emerg Med 2009;27:632. 10.1016/j.ajem.2008.09.014 19497487

[10] Atalla MA, Carangan M, Rozen WM. Re: acute traumatic appendicitis following blunt abdominal trauma. ANZ J Surg 2010;80:572–3. 10.1111/j.1445-2197.2010.05390.x 20795985

[11] Toumi Z, Chan A, Hadfield MB, Hulton NR. Systematic review of blunt abdominal trauma as a cause of acute appendicitis. Ann R Coll Surg Engl 2010;92:477–82. 10.1308/003588410X12664192075936 20513274 PMC3182788

[12] Goldman S, Canastra N, Genisca A. Appendicitis following blunt abdominal trauma: an illustrative case. R I Med J (2013) 2022;105:37–8. 35349619

[13] Bouassria A, Ibn Majdoub K, Yazough I, Ousadden A, Mazaz K, Taleb KA. Traumatic appendicitis: a case report and literature review. World J Emerg Surg 2013;8:31. 10.1186/1749-7922-8-31 23937952 PMC3750490

[14] Paschos KA, Boulas K, Liapis A, Georgiou E, Vrakas X. Traumatic appendicitis in minor blunt abdominal injury. Emerg Med Australas 2012;24:343–6. 10.1111/j.1742-6723.2012.01557.x 22672177

[15] Ahmed ST, Ranjan R, Saha SB, Singh B. Traumatic appendicitis misdiagnosed as a case of haemoperitoneum. BMJ Case Rep 2014;2014:bcr2013202082. 10.1136/bcr-2013-202082 PMC4009847 24759158

[16] Cobb T. Appendicitis following blunt abdominal trauma. Am J Emerg Med 2017;35:1386.S0735-6757(17)30505-3. 10.1016/j.ajem.2017.06.051 28673696

[17] Khilji MF, Zia Ullah Q. Seat belt compression appendicitis following motor vehicle collision. Case Rep Emerg Med 2017:8245046. 10.1155/2017/8245046 28337350 PMC5350481

[18] Zvizdic Z, Pasic-Sefic I, Vranic S. Acute perforated appendicitis after blunt abdominal trauma: a report from a 7-year-old. Am J Emerg Med 2020;38:408.S0735-6757(19)30596-0. 10.1016/j.ajem.2019.158447 31685305

[19] Toffaha A, Al-Yahri O, Hijawi Z, Al-Mudares S, Al-Tarakji M, Shahid F, et al. Appendicitis secondary to trauma following a camel kick. Case Report and Review of Literature Case Rep Surg 2021;2021:6667873. 10.1155/2021/6667873 PMC7808804 33505757

[20] Chalh O, El Haddad S, Choayb S, Allali N, Chat L. Traumatic appendicitis in children. Glob Pediatr Health 2021;8:2333794X21992168. 10.1177/2333794X21992168 PMC7868464 33614856

[21] Salinas-Castro KJ, Mejía-Quiñones V, Zúñiga-Londoño NY. Acute appendicitis after closed abdominal trauma: a case report. Radiol Case Rep 2023;18:631–4. 10.1016/j.radcr.2022.10.088 36471733 PMC9719008

