# Supplementary material for: Traumatic appendicitis is probably not real: an illustrative analysis of coincidental occurrences in nature
Source: Trauma Surg Acute Care Open. 2023 Mar 15;8(1):e001093. doi: 10.1136/tsaco-2023-001093 (PMC10030918; doi:10.1136/tsaco-2023-001093)
Supplement: Supplementary data [file tsaco-2023-001093supp001.pdf]

```perl
#!/usr/bin/env perl

use 5.032;
use strict;
use warnings;

my $population   = 67_100_000;    # UK population
my $appendix_inc = 151;           # incidence of 151 per 100_000 per year
my @trauma_inc_array =
  ( 1, 10, 100, 1000, 10000 );    # 1 in whatever number specified here

say "Trauma_incidence \t Run \t No_of_appendix \t No_of_trauma \t
No_of_traumatic_appendicitis ";

for my $trauma_inc (@trauma_inc_array) {

    for my $run ( 1 .. 1000 ) {
        my $appendix_total           = 0;
        my $trauma_total             = 0;
        my $traumatic_appendix_total = 0;

        for ( 1 .. $population ) {
            my $appendix_prob = int( rand(100_000) ) + 1;
            my $trauma_prob   = int( rand($trauma_inc) ) + 1;
            my $appendix_day  = int( rand(365) ) + 1;
            my $trauma_day    = int( rand(365) ) + 1;

            $appendix_total += 1 if ( $appendix_prob <= $appendix_inc );
            $trauma_total   += 1 if ( $trauma_prob == 1 );

            if (    ( $appendix_prob <= $appendix_inc )
                and ( $trauma_prob == 1 )
                and ( $appendix_day - $trauma_day >= 0 )
                and ( $appendix_day - $trauma_day <= 3 ) )
            {
                $traumatic_appendix_total += 1;
            }
            elsif ( ( $appendix_prob <= $appendix_inc )
                and ( $appendix_day <= 3 ) )
            {
                $trauma_prob = int( rand($trauma_inc) ) + 1;
                if ( $trauma_prob == 1 ) {
                    my $trauma_day = int( rand(365) ) + 1;
                    if (    ( $appendix_day - $trauma_day + 365 >= 0 )
                        and ( $appendix_day - $trauma_day + 365 <= 3 ) )
                    {
                        $traumatic_appendix_total += 1;
                    }
                }
            }
            else {
                next;
            }
        }
        say $trauma_inc, "\t", $run, "\t", $appendix_total, "\t",
          $trauma_total, "\t", $traumatic_appendix_total;

    }
}
```
